# Sonication-Assisted Production of Fosetyl-Al Nanocrystals: Investigation of Human Toxicity and In Vitro Antibacterial Efficacy against *Xylella fastidiosa*

**DOI:** 10.3390/nano10061174

**Published:** 2020-06-16

**Authors:** Francesca Baldassarre, Giuseppe Tatulli, Viviana Vergaro, Stefania Mariano, Valeria Scala, Concetta Nobile, Nicoletta Pucci, Luciana Dini, Stefania Loreti, Giuseppe Ciccarella

**Affiliations:** 1 Biological and Environmental Sciences Department, UdR INSTM of Lecce University of Salento, Via Monteroni, 73100 Lecce, Italy; viviana.vergaro@unisalento.it; 2 Institute of Nanotechnology, CNR NANOTEC, Consiglio Nazionale delle Ricerche, Via Monteroni, 73100 Lecce, Italy; concetta.nobile@nanotec.cnr.it (C.N.); luciana.dini@uniroma1.it (L.D.); 3 Council for Agricultural Research and Economics, Research Centre for Plant Protection and Certification of Rome, 00156 Rome, Italy; giuseppe.tatulli@hotmail.it (G.T.); valeria.scala@crea.gov.it (V.S.); nicoletta.pucci@crea.gov.it (N.P.); stefania.loreti@crea.gov.it (S.L.); 4 Biological and Environmental Sciences Department, University of Salento, Via Monteroni, 73100 Lecce, Italy; stefania.mariano@unisalento.it; 5 Department of Biology and Biotechnology “Charles Darwin”, University of Rome “La Sapienza”, Piazzale Aldo Moro 5, 00185 Roma, Italy

**Keywords:** ultrasonics, fosetyl-Al, chitosan, nanopesticides, *Xylella fastidiosa*, nanotoxicity

## Abstract

Recently, there is a growing demand in sustainable phytopathogens control research. Nanotechnology provides several tools such as new pesticides formulations, antibacterial nanomaterials and smart delivery systems. Metal nano-oxides and different biopolymers have been exploited in order to develop nanopesticides which can offer a targeted solution minimizing side effects on environment and human health. This work proposed a nanotechnological approach to obtain a new formulation of systemic fungicide fosetyl-Al employing ultrasonication assisted production of water dispersible nanocrystals. Moreover, chitosan was applicated as a coating agent aiming a synergistic antimicrobial effect between biopolymer and fungicide. Fosetyl-Al nanocrystals have been characterized by morphological and physical-chemical analysis. Nanotoxicological investigation was carried out on human keratinocytes cells through cells viability test and ultrastructural analysis. In vitro planktonic growth, biofilm production and agar dilution assays have been conducted on two *Xylella fastidiosa* subspecies. Fosetyl-Al nanocrystals resulted very stable over time and less toxic respect to conventional formulation. Finally, chitosan-based fosetyl-Al nanocrystals showed an interesting antibacterial activity against *Xylella fastidiosa* subsp. *pauca* and *Xylella fastidiosa* subsp. *fastidiosa*.

## 1. Introduction

### 1.1. Phytopathogens Control Strategies: The Cases of Xylella fastidiosa and Fosetyl-Al

In the recent years, increased investigations have been undertaken from academy and industry research for innovative and safe solutions to control phytopathogens diffusion. Agrochemicals application is crucial in agriculture preventing crops loss and improving productivity. Degradation processes such as biodegradation, photodegradation and hydrolysis affect conventional application of most pesticides, which are mostly lost in the environment reaching the target only at 0.1% [1]. Therefore, pesticides have greatly influenced the quality of food, air, ground water and surface water causing various environmental problems compromising human and animal health [2]. The active substance for plants treatments must be proven safe for human health, animals and environment including their residues in food. The European Union (EU) regulates precisely the placing on the market and the maximum residue levels (MRLs) in food of plants protection products, including biocidal. Regulation (EC) No 1107/2009 of the European Parliament and of the Council of 21 October 2009 concerning the placing of plant protection products on the market and repealing Council Directives 79/117/EEC and 91/414/EEC, sets the procedures for pesticides authorization, involving Member States and European Food Safety Authority (EFSA). The European regulation asserts that plants protection products are able to: (i) protecting or preventing plants against harmful organisms (e.g., fungicides, insecticides); (ii) influencing plants growth (e.g., hormones); (iii) preserving plant products; (iv) preventing growth or destroying undesired plants or parts of plants (herbicides). Furthermore, biocidal products authorization is regulated in order to ensure a high protection of environment and humans health (Regulation (EU) 528/2012). The European Commission (EC) fixes the MRLs, which are the highest levels of pesticides residues could be present in food or feed assuring consumers safety. These parameters for each product and each crop are resumed in a database on the Commission website (Pesticides EU-MRLs Regulation (EC) No 396/2005). This risk assessment is provided by EFSA. A substantial monitoring work involves the cooperation of Member States and EFSA: National authorities analyze pesticide residues on more than 75,000 food samples each year and send the results to EFSA that publishes a report on which European Commission decisions are based (www.efsa.europa.eu). European Commission promotes the sustainable crops protection strategies via different routes through the common agricultural policy (CAP). Agrochemicals and antibiotics have been severely downsized in organic farming (EU regulation 834/2007 on organic production and labelling of organic products and EU regulation 889/2008 on rules governing organic production, labelling and control). The use of the antibiotic streptomycin in agriculture was banished in 2004 and EU is working to reduce the use of copper (EUR-Lex 32004D0129, Reg UE 2018/1981). However, these latter represent the main control tools for phytopathogenic bacteria, other than preventive measures. Furthermore, bacteria resistance mechanisms and their quick diffusion, also caused by globalization, worsen the situation. In this context, the unexpected arrival of *Xylella fastidiosa* subsp. *pauca* (*Xfp*) in the Salento peninsula of Italy has created an unprecedented emergency. The quarantine bacterium *Xylella fastidiosa* subsp. *fastidiosa* (*Xff*) has been known as the cause of the destructive Pierce’s disease since 1978 in the USA [3]. However, *X. fastidiosa* had never previously established in the EU territory, until the onset of the olive quick decline syndrome (OQDS) associated to *Xfp*, that severely compromised the olive oil industry and nursery trade that represent primary resources for the Mediterranean areas [4]. Currently the management of the infected areas aims to control *Xfp* diffusion through bacterial vector multiplication (laboratory and field trials, agricultural practices involving herbicides and insecticides application), while there is no cure for the infection [4]. Conventional pesticides formulations have important drawbacks in relation to efficacy and toxic effects [2]. Several active substances have poor water solubility, that constrains organic solvents use, low bioavailability and toxicity toward non target organisms. Botanical pesticides have important drawbacks such as slow action and low toxic activity [5,6]. Therefore, there is a great need to explore alternative methods to contrast vectors and phytopathogens preventing side effects on human health and environment. Many works have investigated new pesticides formulations in order to improve efficacy reducing environmental contamination [7,8]. The principal approach is to develop existing agrochemicals reformulations due to high costs of novel active substances design [9]. EC regulation provides a list of potential active substances candidates, both chemical and not, which could replace products for plants protection. The analysis of these candidates must be carried out by EU countries and stakeholders. The output is a comprehensive database that now contains 77 candidates for substitution (“Ad-hoc study to support the initial establishment of the list of candidates for substitution as required in Article 80(7) of Regulation (EC) No 1107/2009”-09.07.2013). Furthermore, Regulation (EC) No 1107/2009 could, extraordinarily, authorize the Member State to be able to place, on the market, a product to contrast an emergency without a solution. Field trials to manage the disease caused by *Xfp*, were carried out in infected area in southern Italy. Notably, different elicitors of plant resistance, including fosetyl-Al, were tested without any beneficial impact in reducing symptoms caused by *Xfp* [10]. Fosetyl-Al is a systemic fungicide that has been also applied against bacterial diseases. A recent work proposed the role of fosetyl-Al in the control of the colonization of pear and cherry leaf surfaces by *Pseudomonas syringae* [11] and for the control of the bacterial canker of kiwifruit caused by *P. syringae pv. actinidiae* (Brunetti and Pilotti, personal communication). Fosetyl-Al has been registered in several European countries for fire blight control, caused by *Erwinia amylovora* [12]. Innovative approaches were recently investigated against *X. fastidiosa*. Chitosan coating and hyaluronan/chitosan nanofilms have been investigated to control the adhesion and growth of Gram negative pathogens, including *X. fastidiosa* cells [13,14]. Synthetic biodegradable polymers, such as polyethylene, polyvinyl alcohol, ε-caprolactone, polyester, polyurethanes and natural ones, such as sodium alginate, carboxymethyl cellulose, starch, chitosan and pectin are exploited to entrap and efficiently release different antimicrobial substances due to their bio- and eco-compatible and bioadhesive proprieties [15]. In particular, chitosan has been investigated in a wide spectrum of drug delivery systems including pharmaceutical, food industry and agriculture applications thanks to its biocompatibility, antibacterial and plant-immunity eliciting properties [16,17,18]. The antimicrobial efficacy of chitosan is known for a broad range of pathogens [19,20]; in vitro biocidal activity [21] and an in-field efficacy [22] was observed for *P. syringae pv*. *actinidiae*. The activation of a defense responses was reported in *P. syringae pv*. *actinidiae* artificially infected kiwi plants [23]. Recently, new nanotechnological tools were proposed to control *X. fastidiosa*, investigating CaCO_3_ nanocarriers [24,25] interaction with pathogen cells and olive plants [26]. CaCO_3_ nanocrystals evidences have been proved the strategic role of nanopesticides to potentially control *X. fastidiosa* pathogen [26].

### 1.2. Nanopesticides and Sonication Technology

Nanotechnologies in agriculture allow to achieve innovation request developing the so-called nanopesticides and nanofertilizers thanks to the exploitation of nanomaterials, biopolymers and delivery systems [27,28]. Phytotoxicity and human side effects could be contained. The nanoformulation is able to significantly influence sorption and degradation phenomena determining pesticide fate and environmental impact [29]. Different micro- and nano-capsules have been formulated and commercialized for pesticides encapsulation and smart delivery [30,31]. Various types of nanomaterials are used as antibacterial agents aiming the substation of chemical pesticides in crops science [32]. The most used among these novel antimicrobial products are the metal oxides, copper and silver nanoparticles [33,34,35]. These nanomaterials have shown relevant nanotoxicology effects toward animals, human cells and plants [36,37]. Many fungicides nano-formulations, including capsules and biopolymers shell, have been developed and investigated for their improvement of target site diffusion easing phytotoxic effects [38,39]. Therefore, the research of new antimicrobial nanomaterials as well as nanoformulations is still a current topic. Sonication is a very effective method to fabricate or manipulate nano- and bio-materials in many applications [40]. High power ultrasounds can break-down chemical bonds depolymerizing macromolecules, downsizing particles to nanoscale, making nanoemulsions, extracting bioactive substances from different matrices and so on, in research laboratories, in pharmaceutical and agrifood industries [40,41,42,43]. Sonochemistry was recently investigated for synthesis of bioactive nanostructures [44], different nanocarriers such as niosomes [45], liposomes [46], nanoemulsions [47], solid lipid nanoparticles [48], metal based systems [49], polymeric particles [50], nanoclusters [51] and nanocomposites [43]. Sonication processes were mainly performed to extract or degrade pesticides in order to support analysis and decontamination activities [52,53]. Nanocarriers and nanoemulsions were successfully prepared by sonication in order to enhance agrochemicals plants uptake and transport [54]. Furthermore, sonochemical synthesis was recently applied to produce different nanomaterials as detection platforms for pesticides [55]. 

The present work has been provided sonication process to develop a new formulation of fosetyl-Al antimicrobial agent. The nanoformulation was obtained employing chitosan coating and it was characterized in order to investigate crystals stability. Antibacterial in vitro tests were performed to verify effect on *Xylella fastidiosa*. Moreover, in vitro cytotoxicity tests were carried out to explore the potential safe use of this novel formulation on field phytopathogens control. 

## 2. Materials and Methods

### 2.1. Materials

All reagents were used without further purification. Chitosan (CH) medium molecular weight and glacial acetic acid were purchased from Sigma–Aldrich (Milano, Italy). Aliette by Bayer was applicated as commercial formulation of fosetyl-Al (Fos). For cells culture and experiments, the following reagents are used: Dulbecco’s Minimum Essential Medium (DMEM) (Cambrex, Verviers, Belgium), fetal calf serum (FCS), glutamine (Cambrex, Verviers, Belgium), penicillin and streptomycin solution (Cambrex, Verviers, Belgium), Spurr resin (TAAB, Berks, UK), tolulidine blue (Sigma-Aldrich, Milano, Italy), cacodilate buffer and 3-(4,5-dimethylthiazol-2-yl)-2,5-diphenyltetrazolium bromide salt (MTT) (Sigma-Aldrich, Milano, Italy). For antibacterial test: Gentra Puregene Yeast/Bact. Kit was purchased from Qiagen (PL Venlo, The Netherlands), SYBR select master mix from Applied Biosystem (Foster City, CA, USA).

### 2.2. Sonication Assisted Fabrication of Fosetyl-Al Nanocrystals

A solution of fosetyl-Al commercial formulation was prepared at 1 g/L and sonicated for 30 min at 150 Watt using High Intensity Ultrasonic Processor-750 Watt Model (SONICS & MATERIALS, Newtown, CT, USA). Chitosan medium MW was used for the preparation of chitosan-based nanoformulation. The 0.5% (*w*/*w*) chitosan solution was prepared by dissolving polymer in 0.1% glacial acetic acid and stirred at room temperature for 3 min at 18.000 rpm with T25 digital Ultra Turrax (IkaLab, Milano, Italy). Sonication conditions are the same as already described. 

### 2.3. Characterization of Fosetyl-Al Nanocrystals

The morphological analysis of fosetyl-Al nanocrystals (nanoFos) and chitosan-based fosetyl-Al nanocrystals (CH-nanoFos) was conducted with transmission electronic microscopy (TEM) and high resolution scanning (transmission) electron microscopy (HRS(T)EM) or (SEM/STEM). Samples preparation follows the same protocol for the two techniques: A drop (10 μL) of each sample solution was placed on a standard carbon-coated TEM Cu-grid and let the solvent to dry at room temperature. Conventional TEM and SEM/annular dark field (ADF)–STEM imaging of the as-prepared sample grids were recorded, respectively, by a TEM microscope JEOL JEM 1400Plus (Peabody, MA, USA), equipped with a bottom-view camera Gatan Orius SC600 (Pleasanton, CA, USA) and a LaB_6_ filament—source operating at an accelerating voltage of 80 kV, and by a FE-SEM microscope Carl Zeiss Merlin (Oberkochen, Germany), equipped with a Gemini II column, with a complete high angle annular and annular dark field/bright field (HAADF/BF) STEM detector system, and with FEG source operating at an accelerating voltage of 20 kV and with short exposure time of a few seconds to minimize sample damages.

Hydrodynamic diameter and ζ-potential measurements were performed through the instrument Nano ZS90 (Malvern Instruments, Malvern, UK) with an appropriate dilution of the samples in water at room temperature. Clear disposable ζ-potential cells (1 cm path length) and cuvettes were rinsed with distilled water, followed by deionized water prior to sample loading. The ζ-potential of the samples is reported as the mean value of five measurements; each measurement derived from five different readings to establish measurement repeatability. Hydrodynamic diameter is reported as average data of three experiments. 

Characterization has been repeated for each nanocrystals batch to asses production reproducibility.

### 2.4. Toxicological Study

#### 2.4.1. Cell Culture

HaCat cells were cultured in DMEM supplemented with 10% FCS, 2 mML-glutamine, 100 IU/mL penicillin and streptomycin solution in a 5% CO_2_ humidified atmosphere at 37 °C. Cells were maintained in 75 cm^2^ flasks (concentration ranged between 2 × 10^5^ and 1 × 10^6^ cells/mL) by passage every 3–4 days, when the culture reached approximately 80% confluence; cells were then seeded in 9.6 cm^2^ multiwells (for MTT test and bright field optical microscopic observation) or in 75 cm^2^ flasks (for TEM analysis) and held in a 37 °C, 5% CO_2_ incubator for at least 24 h before processing the cells further.

#### 2.4.2. MTT Assay

The citotoxicity of Fos, nanoFos and CH-nanoFos at different times (18 h, 24 h and 48 h) and concentrations (1000 µg/mL, 100 µg/mL and 10 µg/mL) was evaluated by MTT assay. Incubation of cells with DMEM culture medium alone was used as negative control. At fixed times after treatment, the culture medium was discharged, the cells were washed two times with phosphate-buffered saline (PBS) and fresh culture medium containing 1 mg/mL of 3-(4,5-dimethylthiazol-2-yl)-2,5-diphenyltetrazolium bromide salt was added to each well. After 2 h incubation at 37 °C in a 5% CO_2_ humidified atmosphere, MTT was reduced to formazan salt, a dark insoluble product, by the mitochondrial reductase of vital cells. When formazan salts were dissolved in dimethylsulfoxide (DMSO), led to a violet solution whose absorbance was measured with Ultrospec 4000 UV-visible spectrophotometer (Pharmacia Biotech, Stockholm, Sweden) at 570 nm. Viability was expressed as percentage of the relative growth rate (RGR) by the equation:

RGR = (Dsample/Dcontrol) × 100(1)

where Dsample and Dcontrol are respectively the absorbance of the test samples and the negative controls. Morphological studies of treated and non-treated cells were carried out using inverted microscope Eclipse 80i (Nikon, Tokyo, Japan).

#### 2.4.3. Ultrastructural Analysis

Two concentrations (1000 µg/mL, 100 µg/mL) of Fos, nanoFos and CH-nanoFos for 24 h were chosen to perform the ultrastructural analysis of HaCat cells. Cells were fixed in sodium cacodilate buffer 0.1 M, pH 7.2, containing 2.5% glutaraldehyde, for 2 h at 4 °C. Samples were then washed three times for 10 min in sodium cacodilate buffer, postfixed in 1% osmium tetraoxide and washed twice for 10 min in sodium cacodilate buffer 0.1 M, pH 7.2. Samples were stained with 0.5% uranyl acetate o.n. at 4 °C. Samples were washed with distilled water and dehydrated in a graded series of ethanol, from 30% to 100% and then, were embedded in Spurr resin and polymerized for 2 days at 60 °C. Semithin sections of 500 nm in thickness were cut using an ultramicrotome PowerTome PT-PC (RMC, München, Germany), stained with 1% tolulidine blue and observed under microscope to determine the presence of the sample. Ultrathin sections of 50 nm in thickness were cut from selected blocks. Sections were picked up in 200 mesh copper grids and examined under a Hitachi HT7700 transmission electron microscope (Tokyo, Japan) at 75 kV.

### 2.5. Antibacterial Activity

#### 2.5.1. In Vitro Growth Assay

*Xylella fastidiosa* subsp. *pauca* strain De Donno (CFBP 8402) (*Xfp*) and *Xylella fastidiosa* subsp. *fastidiosa* (*Xff*) strain Temecula1 were grown in PD2 agar medium for 15–20 days at 28 °C, scraped off, resuspended in PD2 broth and grown to 10^6^ CFU mL^−1^. The concentration was spectrophotometrically measured at A600 = 0.8 OD [56]. Bacterial inoculum (60 µL) was transferred in 6 mL of PD2 broth alone, as control, or in PD2 broth supplemented with Fos, nanoFos and CH-nanoFos. For a preliminary assessment the compounds were used in a single administration at time 0, and then supplemented three times (day 0, day 3 and day 9) (100 µg/mL, 10 µg/mL and 1 µg/mL final concentrations) in order to evaluate the effect on planktonic and biofilm growth. Three replications were performed for each dilution of each compound and all experiments were carried out three times (*n* = 9).

#### 2.5.2. Genomic DNA Extraction and Real-Time PCR

Planktonic growth was assessed after 6 and 15 days by real time PCR on genomic DNA (gDNA) against time 0. Genomic DNA was extracted from each bacterial culture (500 µL) using the Gentra Puregene Yeast/Bact. Kit (Qiagen, PL Venlo, The Netherlands) according to the manufacturer’s instructions for Gram-negative bacteria. The real-time PCR was performed according [57] following EPPO PM7/24 (4) using SYBR select master mix for CFX (Applied Biosystem, Foster City, CA, USA). Each DNA sample was analyzed in triplicate.

#### 2.5.3. Cristal Violet Assay

Biofilm growth was evaluated, after 15 days of treatment, according to the method of Zaini et al. [58], with little modifications. PD2 broth was removed, and the tubes were gently rinsed twice with sterile distilled water. Biofilm was stained with 500 μL of 0.01% crystal violet for 20 min. The tubes were first rinsed and gently washed with sterile distilled water and subsequently with 1 mL of absolute ethanol. The adsorbance at 600 nm of the resulting ethanol solution was measured with DeNovix Spectrophotometer DS-11 Fx+ (Denovix Inc., Hanby Building Wilmington, DE, USA). 

#### 2.5.4. Agar Dilution Assay

*Xfp* and *Xff* growth was assessed adding CH-nanoFos-Al to PD2 medium (final concentration of 100 µg/mL, 10 µg/mL and 1 µg/mL) following Bleve et al. 2018. *Xfp* strain De Donno and *Xff* strain Temecula1 were grown in PD2 broth for 7 days at 28 °C and 100 rpm. Then, 10 μL of decimal dilutions from 10^5^ to 10^1^ CFU mL^−1^ of each inoculum were applied to the PD2 agar plates supplemented or not with CH-nanoFos-Al. The agar plates were incubated at 28 °C for 8 days for *Xff* and 20 days for *Xfp*. Four replications were performed for each dilution and each experiment was carried out two times (*n* = 8). All images were captured by using a stereo microscope (Wild Heerbrugg, Heerbrugg, Switzerland) connected to a digital camera (Leica IC80 HD) in the same conditions of bright field illumination. CFU counting and total area analysis was optically quantified, for each image, after correcting image contrast and brightness at the same conditions, by using ImageJ software 1.52v.

#### 2.5.5. Statistical Analysis

Data were analyzed by performing one-way analysis of variance (ANOVA) at the 95% confidence level. Statistical significance of the in vitro analyses was determined by the Student t-test for pairwise comparison of means. All statistical analyses were carried out by using GraphPad Prism 5 Software (GraphPad Software, San Diego, CA, USA). P-values less than 0.05 were considered significant. Data are the mean ± standard deviation (SD) of three independent experiments each done in triplicate.

## 3. Results and Discussion

### 3.1. Preparation and Characterization of Fosetyl-Al Nanocrystals

**Figure 1:**
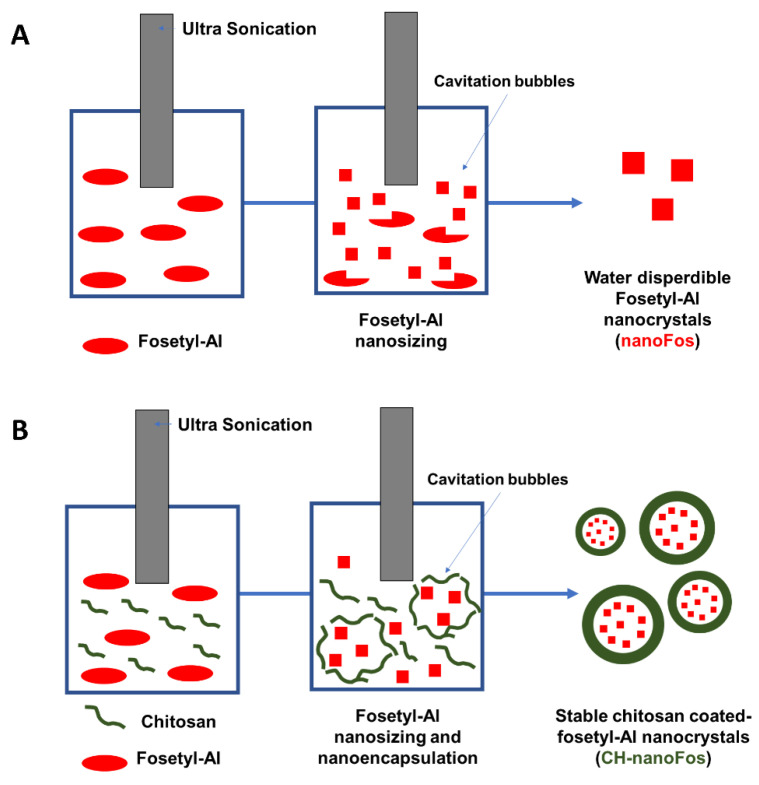
Scheme of ultrasonication-assisted production of fosetyl-Al nanocrystals (nanoFos) (**A**) and chitosan-based fosetyl-Al nanocrystals (CH-nanoFos) (**B**).

A sonication assisted top-down approach was applied for downsizing of poorly water-soluble pesticide fosetyl-Al. Ultrasonication into chitosan solution was performed to coat and stabilize nanocrystals. The outline of nanocrystals synthesis is illustrated in Figure 1. The propagation of ultrasounds into liquid media generate alternating cycles of compression and rarefaction producing vacuum bubbles which accumulate energy and then release it violently. This process, known as cavitation, can trigger and accelerate various chemical reactions including nanocrystals formation. Sonication could be used also in combination with nanoencapsulation methods in order to obtain nanosized and stable delivery systems providing colloids desegregation [59]. Cavitation process is also exploited to improve dissolution rate of poorly soluble drugs, producing a stable and bioavailable nanosuspension and accelerating their dissolution in water [60].

**Figure 2:**
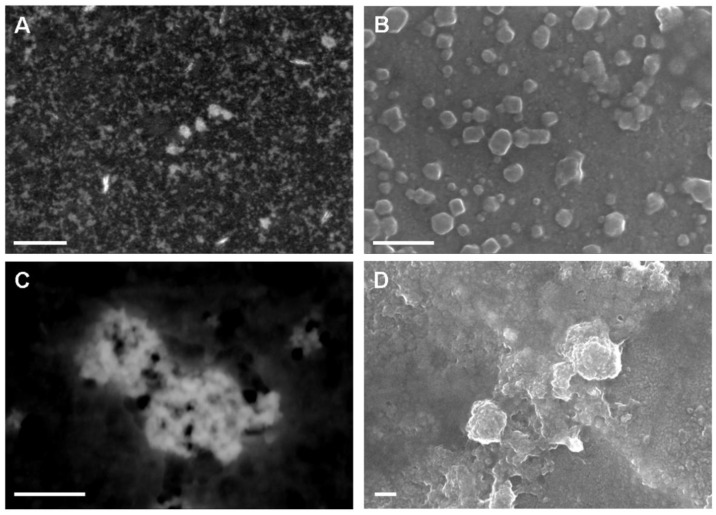
Annular dark field (ADF)-STEM (left) and SEM (right) images of nanoFos (**A**,**B**) and of CH-nanoFos. (**C**,**D**). Scale bar is 200 nm.

The effect of sonication on the morphology of suspended powders in water is clearly shown by electron microscopy characterization in Figure 2. nanoFos appeared with irregular shape and diameter below 100 nm (Figure 2A,B). As shown in Figure 2C,D, chitosan produced colloids of 200–300 nm resulting from the composition of fosetyl-Al nanocrystals. Polymers shell is more evident observing the wrinkled surface of the structures imaged by SEM in Figure 2D and corresponding image–zoom in Figure S1 (Supplementary Materials). The organic halo surrounding a CH-nanoFos colloid can be also observed in the TEM image of Figure S2 (Supplementary Materials). 

This last approach was exploited in order to obtain fosetyl-Al stable nanocrystals. Morphological analysis shown a monodisperse fosetyl-Al nanosuspension following the sonication process. Physical stability was analyzed by DLS (Dynamic Light Scattering) measurement. The Table 1 resumed colloidal stability parameters for nanoFos and CH-nanoFos crystals.

**Figure 3:**
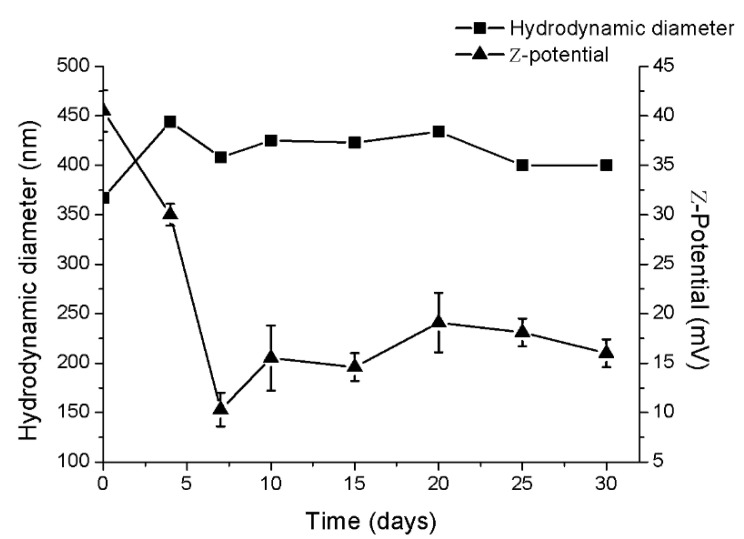
CH-nanoFos colloidal stability: Hydrodynamic diameter and ζ-potential over time.

Measured ζ-potential and size distribution revealed a very different situation from that observed under microscopic analysis. nanoFos showed a very low ζ-potential value anticipating a certain colloidal instability that caused formation of aggregates in water as high hydrodynamic diameter and polydispersity index indicated. The presence of chitosan worked as coating agent and stabilizer providing a high positive ζ-potential and a polydispersity index below 0.5 and a hydrodynamic diameter near to the effective diameter. Polymer could provide steric stabilization and arrested the particle growths which were attributed to the reduction of particles size in water [61,62]. The combination of sonication and layer-by-layer polyelectrolytes assembly has provided many stable nanocolloids of poorly soluble drugs [60,63]. Moreover, the provided electrostatic repulsion has inhibited nanocrystals aggregation over time (see Figure 3). 

There is a reduction in the ζ-potential value to 10–15 mV and a slight increase in the hydrodynamic diameter. A gradual detachment of polymer shell is indicating by positive charges reduction which however did not provide nanocrystals aggregation, as indicated by size distribution. The polydispersity index remained at 0.3. These data confirmed that ultrasonication process provided dissolution in water of Fos producing nanocrystals which were subjected to aggregation and long-term flocculation in aqueous solution, as reveled by physical stability study. Chitosan was chosen as coating agent because of its bioadhesive, biocompatible and antibacterial nature [18]. Polymer provided a superficial positive charge generating colloidal stability in order to exploit the new Fos formulation in the subsequent tests. Surface chemistry also greatly affects cellular uptake efficiency that could define particles biodistribution and therefore their toxicity [64]. Therefore, chitosan coating was exploited not only to stabilize fosetyl-Al nanosuspension but also to reduce its potential toxic response. Toxicological differences of bulk fosetyl-Al, nanoFos and CH-nanoFos were investigated in in vitro studies on human keratinocytes cells line. 

### 3.2. Toxicological Study

**Figure 4:**
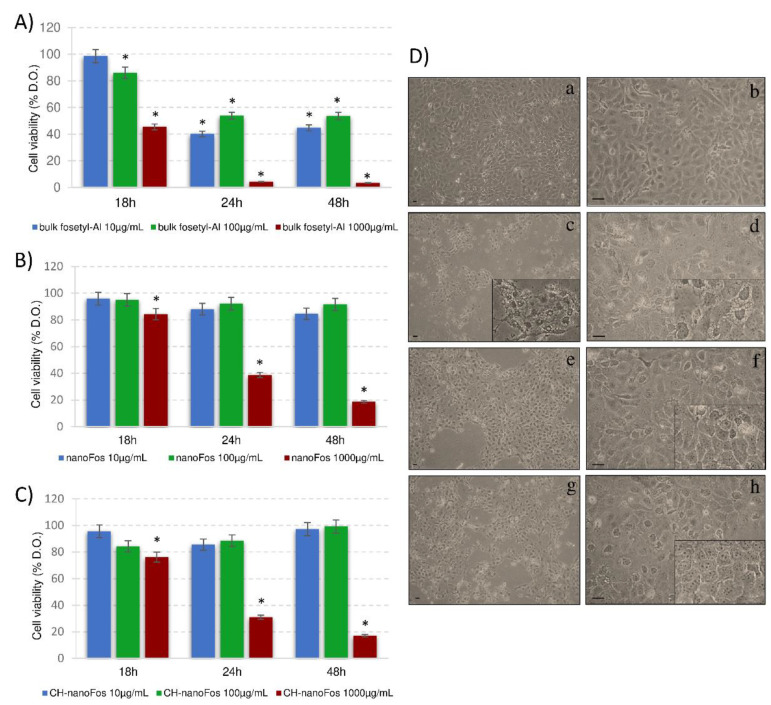
(**A**–**C**), 3-(4,5-dimethylthiazol-2-yl)-2,5-diphenyltetrazolium bromide salt (MTT) assay of (**A**) bulk fosetyl-Al; (**B**) nanoFos; (**C**) CH-nanoFos. The viability measured as indicated in the Methods section represents the values as percentage of the respective control (untreated cells) ones from three independent experiments considered as 100%. Asterisks indicate significant values (*p* ˂ 0.05) from the respective untreated control cells. (**D**) optical microscopic (OM) micrographs show HaCat cells in culture. (**a**,**b**) untreated HaCat cells (100% of viability); (**c**) HaCat cells treated with bulk fosetyl-Al 1000 µg/mL; (**d**) HaCat cells treated with bulk fosetyl-Al 100 µg/mL; (**e**) HaCat cells treated with nanoFos 1000 µg/mL; (**f**) HaCat cells treated with nanoFos 100 µg/mL; (**g**) HaCat cells treated with CH-nanoFos 1000 µg/mL; (**h**) HaCat cells treated with CH-nanoFos 100 µg/mL. Bar = 20 µm.

A consistent use of fungicides is adopted because of many harmful fungal pathogens affect plants causing significative crop yield and quality issues. Fungicides adverse effects on human and animal health, soil organisms, including bacteria, have been verified [65,66]. The systemic fungicide fosetyl-Al is degraded rapidly in soil and plant tissues and the consequent accumulation of phosphonate in crops was revealed [67]. The potential nanoformulations effect on nontarget organisms was investigated in order to support the safe assessment for the future large-scale production and application. Here, we reported the effects of bulk Fos and two fosetyl-Al nanoformulations on human keratinocytes cells, HaCat cell line. HaCat cells choice as in vitro model depends on the literature data reporting that the main route of pesticide poisoning is contact and then absorption through the skin following spills, nebulization or disposal of pesticides [68]. Formulations/cells interaction was investigated in terms of cytotoxicity, morphological effects and cells internalization. Tests were performed with three independent experiments (with three technical replicates for each repeated experiment) by using the same batch of cell culture. The MTT results of HaCat cells viability incubated with different concentrations of the three formulations are reported in Figure 4A. Fosetyl-Al reduced the viability of HaCat cells in a concentration and incubation time dependent manner. In particular, the highest concentration (1000 µg/mL) was more toxic than the others already at 18 h of incubation showing a 50% reduction of viable cells. The highest toxicity was observed at 48 h, when almost all cells were not living (97% reduction vs. untreated cells). Lower concentrations significantly decreased of about 50% vs. control cell viability from 24 h. Only the highest concentration (1000 µg/mL) of nanoFos caused a significant cell viability reduction that increased with time of cell treatment (Figure 4B). Conversely, cells incubated with lower concentrations remained viable at any time of treatment, being comparable to untreated cells, thus indicating that cytotoxicity only depends on formulation concentration. The cytotoxicity of the nanoformulations, nanoFos and CH-nanoFos, was only found at the highest concentration and at the longer time of incubation (Figure 4C). Interestingly, at 48 h of treatment an opposite trend was found; lower concentrations induce a slight increase in viability. Nanomaterials have different physic-chemical features from their respective bulk form, since their interaction with biological systems could considerably change. Size of so-called nanodrugs determines their biodistribution in terms of adsorption, distribution, metabolism and excretion [69]. Particles around 100 nanometer could be internalized by cells through pinocytosis unlike the larger particles which are engulfed by phagocytosis [70]. Micrometer particles are more subject to phagocytosis than nanometric particles. Size dependent toxicity, for inorganic nanoparticles such as metal-based nanoparticles, is well documented in the literature, both phytotoxicity and human cells studies [71,72,73]. Morphological analysis data corroborate MTT assay findings. Observing optical microscopic (OM) images in Figure 4D, it is evident that cell morphology changed accordingly to the decrease of HaCat cells viability. After 24 h of incubation the percentage of living and still adherent cells decreases dramatically with the highest concentration of bulk Fos (Figure 4D,c), and to a lesser extent, but still significant, with a high dose of the two nanoformulations (Figure 4D,e–g). Cell morphology features of toxicity corresponded to floating cells, cell shrinkage and blebbing (Figure 4D,c). Suffering cells, mainly those treated with lower concentration showed an abundant presence of vacuoles and damaged structures (Figure 4D,d). NanoFos and CH-nanoFos at lower concentration no had effects on cell viability (Figure 4D,f–h). 

**Figure 5:**
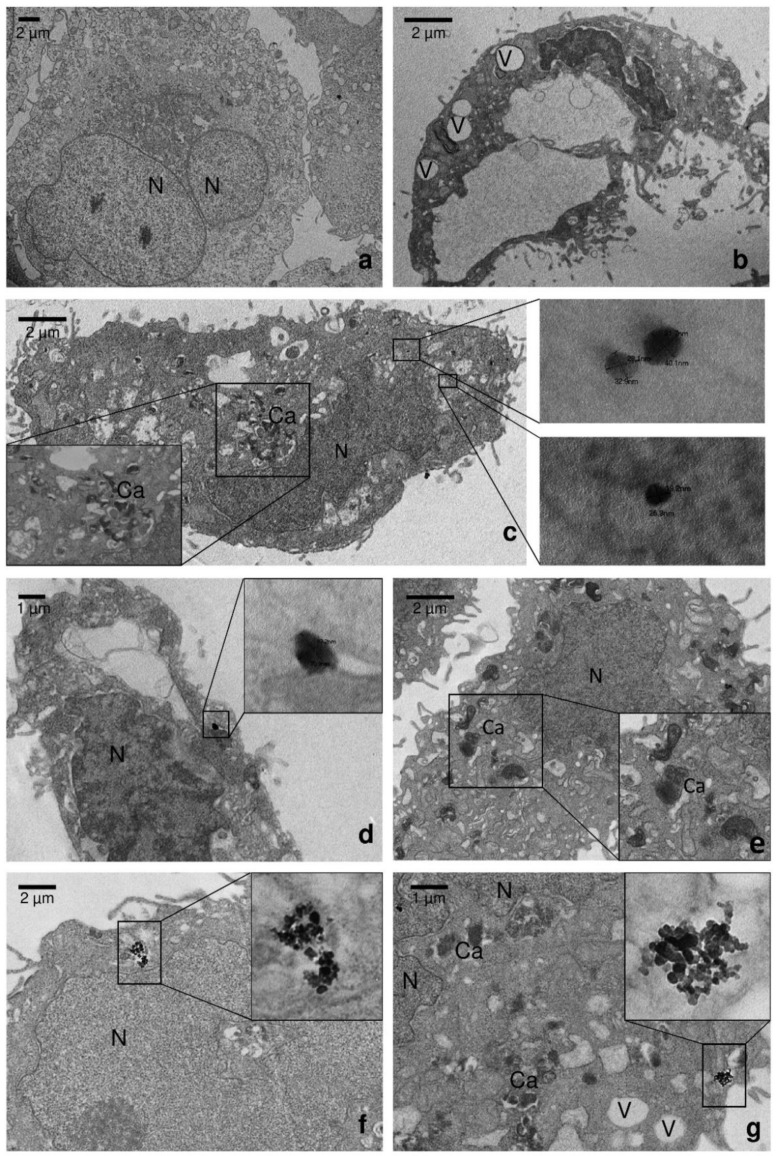
TEM ultrastructural analysis of ultrathin sections of (**a**) untreated cells and cells exposed for 24 h to 1000 µg/mL of: (**b**) bulk fosetyl-Al; (**c**) nanoFos; (**d**) CH-nanoFos; and 100 µg/mL of: (**e**) bulk fosetyl-Al; (**f**) nanoFos; (**g**) CH-nanoFos. N = nuclei; V = vacuoles; Ca = calcifications.

These data agree with most of the works on pesticides. For example, Bakre and Kaliwal demonstrated the toxic effect of two fungicides (carbendazim and copper oxycloride) on HaCat cells and HepG2 cells in term of cell viability, indicating the long exposure to these compounds could lead to lethal effects [74]. In another study, HaCat cells were used to assess the combined effect of low dose of a mixture of three pesticides (lpha-hexachlorocyclohexane, parathion methyl and carbofuran). Results revealed that mixed pesticides were more hazardous for human health, exerting a high toxicity with respect to individual ones [75]. Deleterious consequences have been observed also with other organophosphate pesticides (such as omethoate and methamidophos) inducing different levels of cyto and genotoxic effects in human cell lines [76]. There are still few studies concerning toxic effects of commercial pesticides micro and nanopolymeric formulations in order to verify the influence of size on products safety [77]. The toxicity of the pesticide to the skin depends on the duration of exposure, the formulation of the pesticide and the contaminated part of the body [78]. One study has been published relating to fosetyl-Al toxicity on human cells. Andersen et al. have tested in vitro 24 pesticides for agonistic and antagonistic effects in estrogen and androgen assays and for effects on aromatase activity [79]. The effects of the different formulations of fosetyl-Al were also investigated at ultrastructural level. Figure 5 shows TEM micrographs of cells incubated with bulk fosetyl-Al, nanoFos and CH-nanoFos for 24 h. Untreated cells were used as control (Figure 5a). When cells were exposed to bulk fosetyl-Al (1000 µg/mL), they showed evident nuclei disruption, whole cell damages and the presence of big vacuoles (Figure 5b) which seems to be correlated to drastic reduction of viability observed in MTT assay. Another evident alteration that occurs was the presence of calcification remains (Ca) deriving from degenerating mitochondria (Figure 5c,e,g). Cells exposed to high concentration of nanoFos and CH-nanoFos did not show evident damaged or lysed nuclei, but some vacuoles and alteration of cell structure (i.e., mitochondria damage) were observed (Figure 5c,d). Lower concentrations did not lead to evident changes in cell morphology, except for alterations related to mitochondria which appeared like more electron-dense. Interestingly, nanoFos and CH-nanoFos crystals were found inside the cells, predominantly localized in the cytoplasm. Figure 5c,d show magnified regions of nanocrystals, with a diameter of about 30 nm and 80 nm, respectively. At low concentrations, nanocrystals were found in the cytoplasm as aggregates (Figure 5f,g). Particles of bulk fosetyl-Al were no found inside the cells. This is probably due to poor water solubility and low bioavailability of conventional fosetyl-Al. In general, although the toxicity of the bulk materials is even known, it is not yet understood the link between concentration or size and toxicological properties [80]. In this context, the two fosetyl-Al nanoformulations are less toxic than fungicide in bulk form. It is possible that nanosized pesticides have characteristics that make them more biocompatible, at least in low concentrations. Chitosan coating did not seem to influence nanocrystals interaction with human keratinocytes cells. 

### 3.3. Antibacterial Activity

**Figure 6:**
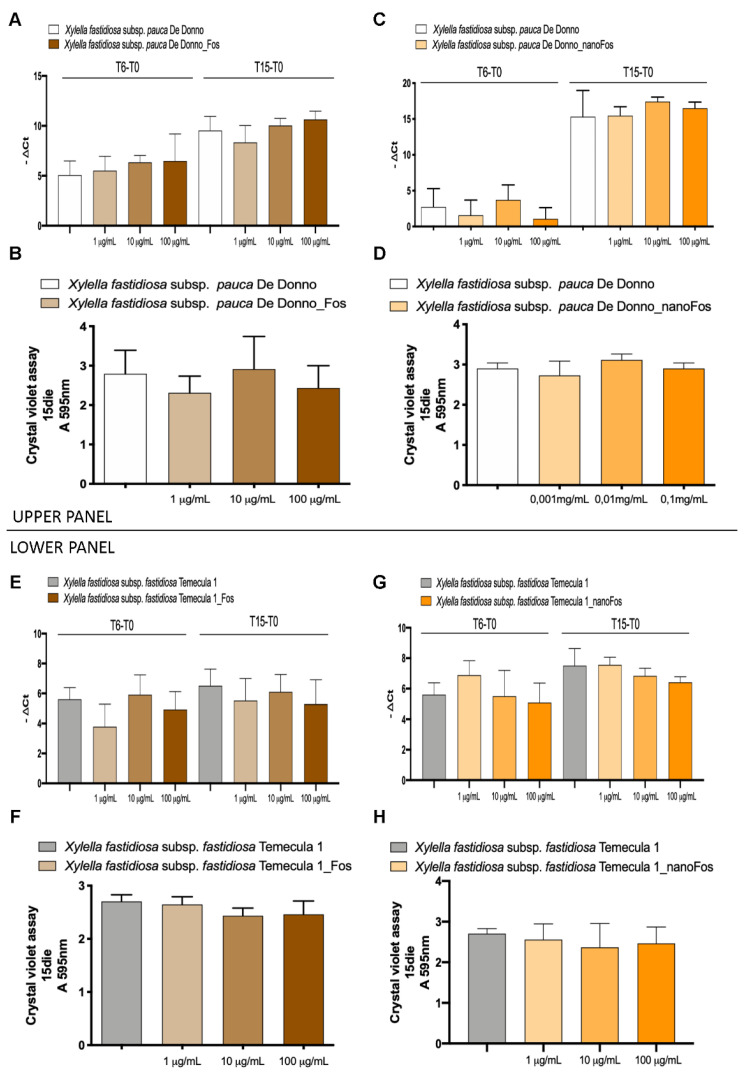
Fosetyl-Al and fosetyl-Al nanoformulations efficacy on *Xfp* strain De Donno and *Xylella fastidiosa* subsp. *fastidiosa* (*Xff*) strain Temecula1 planktonic and biofilm growth. Efficacy of Fos and nanoFos (100 µg/mL, 10 µg/mL and 1 µg/mL) on *Xylella fastidiosa* subsp. *pauca* (Xfp) strain De Donno (**A**,**C**) and *Xff* strain Temecula1 (**E**,**G**) planktonic growth. Products dilutions are reported in the axis; the ordinate reports the -ΔCt value that represents the difference between the Ct value obtained at each time point (6,15 days) and the Ct value at time 0 (T6-T0; T15-T0). Efficacy of Fos and nanoFos (100 µg/mL, 10 µg/mL and 1 µg/mL) on *Xfp* strain De Donno (**B**,**D**) and *Xff* strain Temecula1 (**F**,**H**) biofilm production. The axis reports the products dilutions; the ordinates report the spectrophotometric absorbance of crystal violet at 15 days. Data are expressed as means ± S.D. (*n* = 9, experiment repeated 3 times with different inoculum).

The efficacy of commercial fosetyl-Al and nanoformulations was assessed on *X. fastidiosa* subsp. *pauca* strain De Donno and *X. fastidiosa* subsp. *fastidiosa* strain Temecula1, to verify a direct antibacterial effect at planktonic and/or at biofilm state of the phytopathogen. This bacterium has a fine balance between planktonic and biofilm state; planktonic state is fundamental for pathogen virulence expression while biofilm state, consisting in bacterial aggregate, allows the bacterium spreading to other hosts with the mediation of the insect vectors. To identify the minimal inhibition concentration, three different concentrations of each product, 100 µg/mL, 10 µg/mL e 1 µg/mL, were chosen. The concentration 1000 µg/mL was not evaluated according to results obtained in human toxicology analyses. Preliminary tests carried out with a single administration, at time 0, of Fos, nanoFos and CH-nanoFos, did not show any effect on both planktonic and biofilm growth (data not shown). The planktonic growth was not evaluated by a spectrophotometric measure of the bacterial growth, because the compounds Fos and CH-nanoFos interfered with it. To avoid the interference of the compounds a DNA extraction and real time PCR assay were performed. The Figure 6 shows the results obtained supplementing the compounds at 3 and 9 days post inoculation in PD2 media. In particular, Fos and nanoFos showed no efficacy either on planktonic growth (respectively Figure 6A,E,C,G), or on biofilm production (Figure 6B,D,F,H).

**Figure 7:**
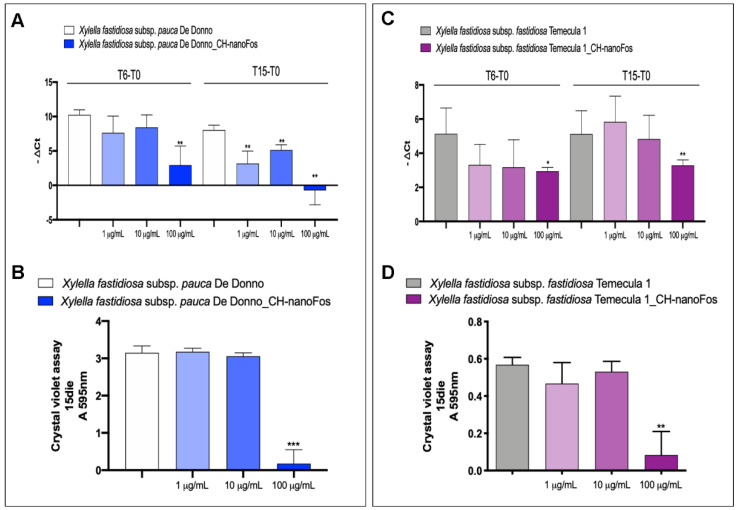
Fosetyl-Al and chitosan-based fosetyl-Al nano-formulations efficacy on *Xfp* strain De Donno (left panel) and *Xff* strain Temecula1 (right panel) planktonic growth and biofilm production. Efficacy of CH-nanoFos (100 µg/mL, 10 µg/mL and 1 µg/mL) on *Xfp* strain De Donno and *Xff* strain Temecula1 planktonic growth (**A**,**C**) and biofilm production (**B**,**D**). CH-nanoFos dilutions are reported in the axis; the ordinate reports, in A/C, the −ΔCt value (difference between the Ct value obtained at 6, 15 days and the Ct value at time 0) and, in B/D, the spectrophotometric absorbance of crystal violet at 15 days. Data are expressed as means ± S.D. (*n* = 9, experiment repeated 3 times with different inoculum; * *p* < 0.05, ** *p* ≤ 0.005, *** *p* = 0.0001 vs. Ctr).

CH-nanoFos, unlike Fos and nanoFos, caused a significant planktonic growth inhibition already after 6 days of treatment at the concentration of 100 µg/mL, in both *Xfp* and *Xff* (Figure 7A,C). After 15 days of treatment, after the third subministration, planktonic *Xfp* growth was inhibited also with 10 µg/mL and 1 µg/mL concentrations (Figure 7A), while *Xff* was not significantly affected (Figure 7C). Biofilm formation of *Xfp* and *Xff*, after 15 days of treatment, was significantly inhibited only at 100 µg/mL concentration (Figure 7B,D). 

**Figure 8:**
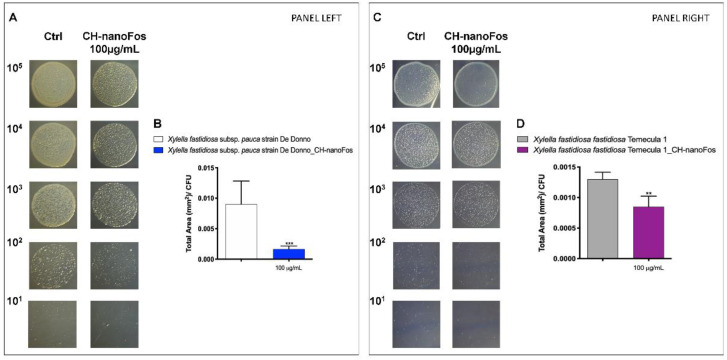
Bacteriostatic/bactericide effect evaluation on *Xfp* strain De Donno (left panel) and *Xff* strain Temecula1 (right panel). (**A**,**C**) 10 μL of decimal dilutions from 105 to 101 CFU mL^−1^ of *Xfp* strain De Donno and *Xff* strain Temecula1 were spotted on PD2 agar plates poisoned or not with CH-nanoFos (100 µg/mL, 10 µg/mL) and incubated at 28 °C (**B**,**D**). The total area and the number of bacterial colonies grown on 102 drops, represented as a ratio between total mass area and CFU grown were counted using ImageJ software. Data are expressed as means ± S.D. (*n* = 8, experiment repeated 2 times with different inoculum; ** *p* ≤ 0.005, *** *p* = 0.0001 vs. Ctr).

To confirm the growth inhibition at 100 µg/mL of CH-nanoFos observed by testing bacterial DNA by real time PCR, this concentration was also evaluated with plating serial dilution of *Xfp* and *Xff* at several concentrations (from 10^5^ to 10^1^ cfu ml ^−1^) on PD2 supplemented or not with CH-nanoFos. As shown in Figure 8A *Xfp* dilutions grows on PD2-CH-nanoFos and appear to be less opaque and whitish than the control, suggesting an inhibitory effect on *Xfp* growth. *Xff* growth reported in Figure 8C seems to be less conditioned from CH-nanoFos than *Xfp*. Image J software was used to count colonies of 10^2^ dilution and their total area. Figure 8B,D shows a significant reduction (both *Xfp* and *Xff*) of the ratio between the total area (expressed in mm^2^) and the CFU counted, on PD2-CH-nanoFos. 

This study showed that fosetyl-Al and nanoFos did not show any bacteriostatic or bactericidal effect in vitro, in line with the results of the field trials [10]. Interesting results were obtained with CH-nanoFos based nanoformulations, designed a stable nanopesticide and exploiting the synergistic effect of biopolymer and fosetyl-Al, which confirmed this assumption by showing in vitro bacteriostatic efficacy against *X. fastidiosa*.

## 4. Conclusions

The environmental and economic damage caused by *Xfp* in Apulia region represented a stimulus in the identification of new compounds and new synthesis methods able to control the bacterium. The research of new Fosetyl-Al formulations, which could be functional against *Xfp* pest and human and environmentally safe, is part of this innovation perspective. This paper focused on the fabrication of highly stable fosetyl-Al nanocrystals made by sonication assisted method. Chitosan was applied as coating agent in order to implement nanosuspension stability and provide synergistic effect toward phytopathogens. Sonication assisted production has successfully carried out fosetyl-Al dissolution providing nanocrystals formation. Moreover, morphological and DLS analysis highlighting the presence of chitosan shell around nanocrystals that provided colloidal stability over time. The nanoformulation significantly reduced toxic effect on human keratinocytes cells with respect the Fosetyl-Al original compound. Although chitosan presence did not seem to influence nanocrystals cyto-compatibility. Ultrastructural analysis by TEM highlighted nanocrystals cells uptake confirming the least cellular damage of nanoformulations respect to bulk pesticide. CH-nanoFos has been shown bacteriostatic effect, unlike fosetyl-Al and nanoFos, on both *Xfp* and *Xff* growth. CH-nanoFos affects the grown and biofilm formation only if added three times after inoculation in liquid media. These results suggested the crucial role of nanopesticide dosage. Moreover, we proved that bacteria grown on PD2 medium supplemented or not with CH-nanoFos reduce significantly the CFU mean area. These results demonstrate the in vitro efficacy of CH-nanoFos on two different *X. fastidiosa* subspecies and represent the first step to understand the role of this nanoformulation in the treatment of *X. fastidiosa*. The study demonstrates that the CH-nanoFos inhibits more promptly and significantly the planktonic growth respect to Fos and nanoFos. These results highlight that nanoformulation alone is not enough but materials engineering strategies, including biomaterials exploitation, is essential in order to maximize biointeractions and effectiveness. In particular, chitosan has played a strategic role in the formation of water-dispersible and stable fosetyl-Al nanosuspension. These features have been very important in order to maximize the interaction with target cells and so the potential applicability in phytopathogens control, as indicated by antibacterial data. On the other hand, the nanoformulation, even without the coating, reduce toxic effects on non-target cells. CH-nanoFos formulation will be further investigated supporting in vitro data with relevance of *in planta* studies. These evidences suggest the possible use of this new nanoformulation in an integrated pest management strategy for an in-filed control of *Xfp*. Therefore, *in vivo* tests are currently in program on plant model *Nicotiana tabacum* to verify the effect of nanoFos and CH-nanoFos on artificially infected plants, exploiting the design and results of in vitro assays (dosage and administration modality). 

## Figures and Tables

**Table 1:** DLS parameters of nanoFos and CH-nanoFos in deionized water (average data of three experiments).

Sample	ζ-Potential	Hydrodynamic Diameter Average	Size Distribution % Intensity	PdI
nanoFos	+0.3 ± 0.4 mV	1200 ± 268 nm	482 ± 215 nm	0.8
CH-nanoFos	+40.5 ± 2.1 mV	367 ± 9 nm	305 ± 63 nm	0.3

## Supplementary Materials

The following are available online at https://www.mdpi.com/2079-4991/10/6/1174/s1, Figure S1: Highly magnified detail of a wrinkled structure in the SEM image of Figure 3D, Figure S2: TEM image of a CH-nanoFos colloid.
