# Supplementary material for: Sonication-Assisted Production of Fosetyl-Al Nanocrystals: Investigation of Human Toxicity and In Vitro Antibacterial Efficacy against Xylella fastidiosa
Source: Nanomaterials (Basel). 2020 Jun 16;10(6):1174. doi: 10.3390/nano10061174 (PMC7353234; doi:10.3390/nano10061174)
Supplement: Supplementary file 1 [file nanomaterials-10-01174-s001.pdf]

nanomaterials

Sonication-assisted production of fosetyl-Al nanocrystals: investigation of human toxicity and *in vitro* antibacterial efficacy against *Xylella fastidiosa*

Supplementary Materials

Francesca Baldassarre [1,2,*,†], Giuseppe Tatulli [3,†], Viviana Vergaro [1,2], Stefania Mariano [4], Valeria Scala [3], Concetta Nobile [2], Nicoletta Pucci [3], Luciana Dini [2,5], Stefania Loreti [3] and Giuseppe Ciccarella [1,2,*]

[1] Biological and Environmental Sciences Department, UdR INSTM of Lecce University of Salento, Via Monteroni, 73100 Lecce, Italy; viviana.vergaro@unisalento.it
[2] Institute of Nanotechnology, CNR NANOTEC, Consiglio Nazionale delle Ricerche, Via Monteroni, 73100 Lecce, Italy; concetta.nobile@nanotec.cnr.it (C.N.); luciana.dini@uniroma1.it (L.D.);
[3] Council for Agricultural Research and Economics, Research Centre for Plant Protection and Certification of Rome, 00156 Rome, Italy; giuseppe.tatulli@hotmail.it (G.T.); valeria.scala@crea.gov.it (V.S.); nicoletta.pucci@crea.gov.it (N.P.); stefania.loreti@crea.gov.it (S.L.)
[4] Biological and Environmental Sciences Department, University of Salento, Via Monteroni, 73100 Lecce, Italy; stefania.mariano@unisalento.it
[5] Department of Biology and Biotechnology "Charles Darwin", University of Rome "La Sapienza", Piazzale Aldo Moro 5, 00185 Roma, Italy
* Correspondence: francesca.baldassarre@unisalento.it (F.B.); giuseppe.ciccarella@unisalento.it (G.C.); Tel.: +39-0832-319207 (F.B.); +39-0832-319810 (G.C.)
† These authors contributed equally to this work.

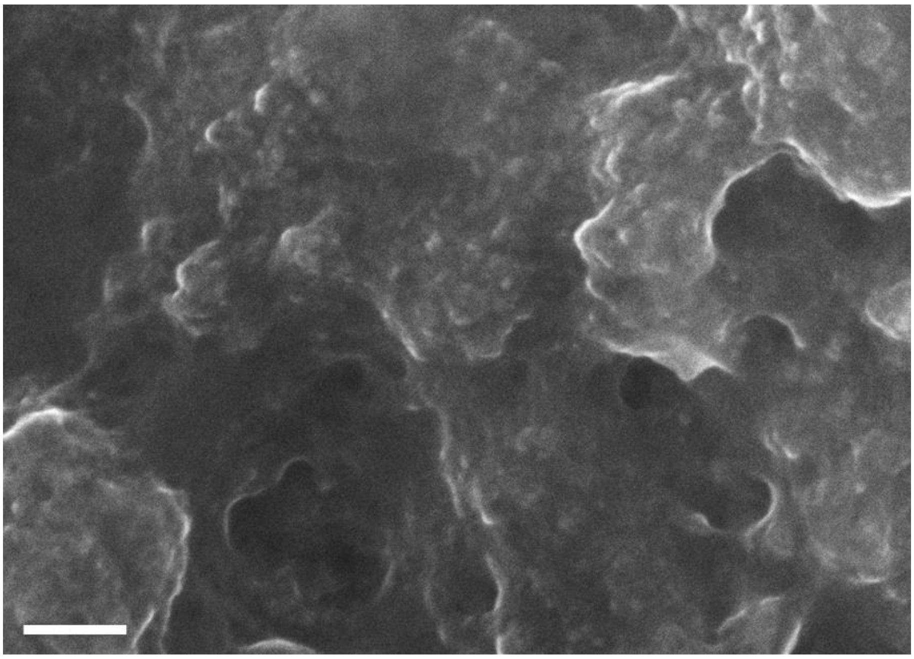

Figure S1. Highly magnified detail of a wrinkled structure in the SEM image of figure 3D.

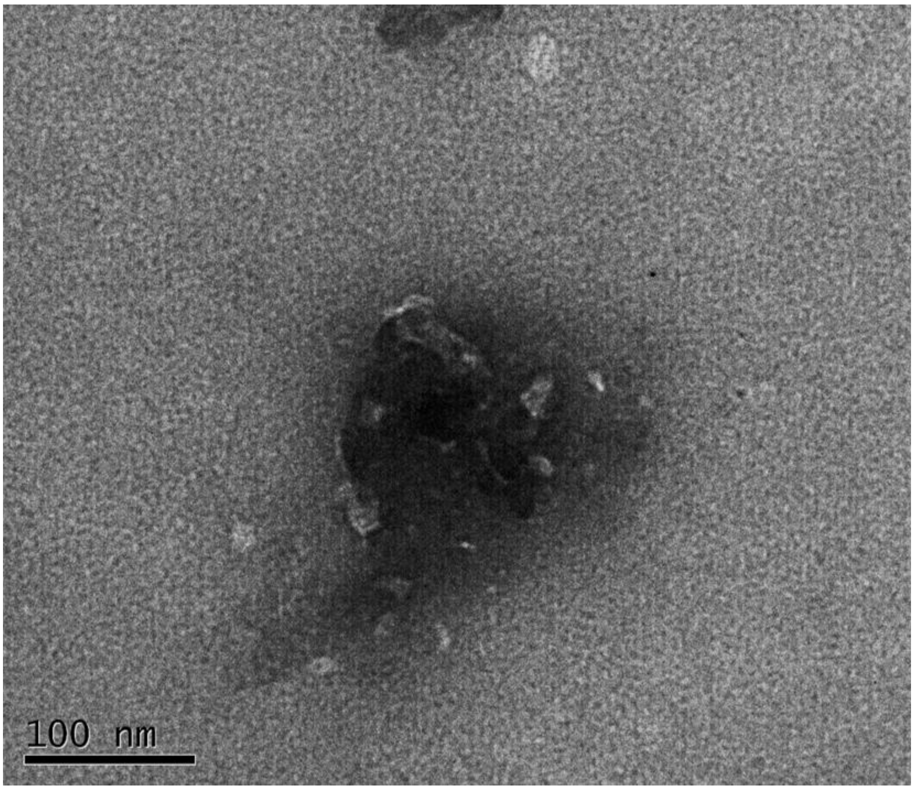

Figure 2. TEM image of a CH-nanoFos colloid.
